# Conversion of Isocyanide to Amine in The Presence of Water and Hg(II) Ions: Kinetics and Mechanism as Detected by Fluorescence Spectroscopy and Mass Spectrometry

**DOI:** 10.3390/ijms21155588

**Published:** 2020-08-04

**Authors:** Anita Adamoczky, Lajos Nagy, Miklós Nagy, Miklós Zsuga, Sándor Kéki

**Affiliations:** Department of Applied Chemistry, Faculty of Science and Technology, University of Debrecen, H-4032 Debrecen, Hungary; adamoczky.anita@science.unideb.hu (A.A.); nagy.lajos@science.unideb.hu (L.N.); miklos.nagy@science.unideb.hu (M.N.); zsuga.miklos@science.unideb.hu (M.Z.)

**Keywords:** steady-state fluorescence, kinetics, isocyanoaminonaphthalenes, HgCl_2_, carbamic acid, carbamate, isocyanate

## Abstract

Aromatic isocyanides including isocyanonaphthalene derivatives have been proven to be very effective fluorescent sensors for the quantification of Hg(II) ions in water. Thus, the reaction of 1,5-isocyanoaminonaphthalene (1,5-ICAN), which is one of the most important members of this family, with water and HgCl_2_ as the oxidation agents, was studied by fluorescence spectroscopy and mass spectrometry in order to get deeper insight into the kinetics and mechanistic details of this complex reaction. The reactions of 1,5-ICAN with water and HgCl_2_ were performed in various water/co-solvent mixtures of different compositions. The co-solvents used in this study were both aprotic solvents including tetrahydrofuran, acetonitrile and N,N-dimethylformamide and protic solvents, such as ethanol and 2-propanol. It was found that in aprotic solvents the conversion of the isocyano group to amino moiety takes place, while in protic solvents the corresponding carbamate (urethane) group is formed in addition to the amino moiety. The variation of the resulting fluorescence intensities versus time curves were described using an irreversible, consecutive reaction model, in which the formation of isocyanate and carbamic acid intermediates, as well as diamino and carbamate (in the case of protic solvents) products were assumed. The formation of these intermediates and products was unambiguously confirmed by mass spectrometric measurements. Furthermore, by fitting the model to the experimental fluorescence versus time curves, the corresponding rate coefficients were determined. It was observed that the overall rate of transformation of the isocyano group to amino moiety increased with the water concentration and the polarity of the co-solvent. It was also supported that formation of diamino and carbamate derivatives in protic solvents takes place simultaneously and that the ratio of the amino to the carbamate function increased with the increasing water concentration. In addition, with an extension, the model presented herein proved to be capable of describing the kinetics of the transformation of 1,5-diisocyanonaphthalene (1,5-DIN) into 1,5-diaminonaphthalene (1,5-DAN) in the mixtures of water/aprotic solvents.

## 1. Introduction

Mercury, either in metallic, covalent or ionic form is one of the most toxic heavy metals found in our environment [1]. Water contaminated with any of these mercury forms poses serious threat to human and animal life, thus the determination of the concentration of Hg-species, e.g., in the form of Hg(II) is of paramount importance [2]. For the selective and sensitive monitoring of the level of Hg(II) in water, numerous methods have been developed so far, spanning from titrimetry [3] through fluorometry [4] to electrochemical [5] or other instrumental detection, such as inductively coupled plasma mass spectrometry (ICP-MS) [6]. Of these, in terms of selectivity and sensitivity, fluorometry appears to be the most cost effective and less time-consuming method. Various fluorometric probes have been designed and applied for Hg(II) determination in water such as those that are based on fluorescence quenching (turn-off) [7], enhancement (turn-on) [8] and recording the fluorescence emissions at two different wavelengths (ratiometric) [9,10,11,12]. The latter, ratiometric method is preferred since it provides self-calibration in a straightforward way and does not suffer from effects such as instrumental efficiencies and probe concentration. One main group of ratiometric fluorophores for detecting Hg(II) contamination involves molecular moieties capable of binding Hg(II) ions reversibly and selectively [11,12,13]. Thus, the sensitivity of these probes largely depends on the stability constant between the Hg(II) ion and the binding site. A second intensively evolving fluorophore family for Hg(II) determination is based on the oxidation/reduction reaction between Hg(II) ions and a specific group of a fluorophore [14,15,16]. Very recently, we have shown that 1,5-isocyanoaminonaphthalene (1,5-ICAN) can react selectively with Hg(II) ions in the presence of water to convert the isocyano to amine group [14]. The conversion of the isocyano moiety to amino group in the presence of Hg(II) ions is accompanied by a considerable blueshift in the observable fluorescence spectrum, thus enabling a very sensitive and selective ratiometric determination of Hg(II) ions in aqueous solution with a limit of detection (LOD) as low as 6 nmol/L. Another efficient ratiometric fluorescent probe based on 1,8-naphthalimide with an isocyano group for the quantification of Hg(II) ions in aqueous medium has been also designed and investigated [15]. Compounds with an isocyano group connected to an aromatic moiety seem to be very promising ratiometric fluorescence probes for Hg(II)-ion detection. However, little is known about the chemistry and kinetics of the conversion of isocyano to amino groups, although the response time and the kinetics of this reaction in different water/co-solvent systems are of great importance in sensing. In this article, we report a detailed kinetic study on the conversion of isocyano to the amino group of 1,5-ICAN in the presence of Hg(II) ions in various water/co-solvent solutions and water-content including co-solvents of both protic and aprotic ones.

## 2. Experimental Materials

We prepared 1-amino-5-isocyanonaphthalene (1,5-ICAN) and 1,5-diisocyanonaphthalene (1,5-DIN) as described previously [17]. Ethanol (EtOH), 2-propanol (*i*PrOH), acetonitrile (ACN), tetrahydrofuran (THF), N,N-dimethylformamide (DMF), potassium hydroxide and chloroform were purchased from Sigma-Aldrich (Darmstadt, Germany). All solvents and reagents were of analytical grade and used as received. 

### 2.1. General Procedure for the Reaction of 1,5-ICAN and/or 1,5-DIN with Water and HgCl_2_

HgCl_2_ was dissolved in the mixture of water and co-solvent (e.g., THF) to obtain a concentration of 1.3 × 10^−3^ mol/L. Three milliliters of this solution was then transferred into a quartz fluorescence cell and thermostated in the fluorimeter. The reaction was started by adding 5 μL ACN solution of the fluorophore to obtain a concentration of 2.5 × 10^−6^ mol/L (with respect to the fluorophore). The water co-solvent ratios were varied during the experiments. 

### 2.2. Monitoring the Reaction Using Steady State Fluorescence Spectroscopy

The fluorescence spectra were recorded at predetermined time intervals using a Jasco FP-8200 fluorescence spectrophotometer equipped with a Xe lamp light source. In all cases the emission spectra were recorded in the range of λ = 350–650 nm at 25 °C, using the excitation wavelength of λ_ex_ = 337 nm, 2.5 nm excitation, 5.0 nm emission bandwidth, and 2000 or 10,000 nm/min scan speed. 

### 2.3. Electrospray Ionization Mass Spectrometry (ESI-MS)

The MS and MS/MS measurements were carried out by a MicroTOF-Q type Qq-TOF MS instrument (Bruker Daltonik, Bremen, Germany). The mass spectra were calibrated externally using the exact masses of cluster ions of sodium trifluoracetate [(NaTFA)_n_+Na]^+^ generated under electrospray conditions. The samples were introduced by a syringe pump (Cole-Parmer Ins. Co., Vernon Hills, IL, USA) at a flow rate of 10 μL/min.

### 2.4. Evaluation of the Kinetic Data

Since the concentration of both the water and the HgCl_2_ was higher by several orders of magnitude than that of the 1,5-ICAN, changes in their concentrations were neglected. The kinetic data obtained from the steady-state fluorescence measurements were evaluated using the irreversible, consecutive “ABC” (*A*→*B*→*C*) reaction model, in which *A*, *B* and *C* represent the initial, the intermediate and the product, respectively. Accordingly, the variation of the concentrations of *A*, *B* and *C* with time *t* can be expressed by Equations (1)–(3):

(1) $$\left[A\right]\left(t\right)={\left[A\right]}_{o}e^{-k_{1}t}$$

(2) $$\left[B\right]\left(t\right)=\frac{k_{1}{\left[A\right]}_{o}}{k_{2}-k_{1}}\left(e^{-k_{1}t}-e^{-k_{2}t}\right)$$

(3) $$\left[C\right]\left(t\right)={\left[A\right]}_{o}\left[1+\frac{k_{1}e^{-k_{2}t}-k_{2}e^{-k_{1}t}}{k_{2}-k_{1}}\right]$$

where *k*_1_ and *k*_2_ are the rate coefficients for the reactions *A*→*B* and *B*→*C*, respectively.

The changes of the fluorescence intensities of *A*, *B* and *C* as a function of time at two different emission wavelengths, one at lower (*I_L_*(*t*)) and the other (*I_H_*(*t*)) at higher λ_em_ value, come as:

(4) $$I_{H}\left(t\right)=p_{A,H}e^{-k_{1}t}+\frac{p_{B,H}k_{1}}{k_{2}-k_{1}}\left(e^{-k_{1}t}-e^{-k_{2}t}\right)+p_{C,H}\left[1+\frac{k_{1}e^{-k_{2}t}-k_{2}e^{-k_{1}t}}{k_{2}-k_{1}}\right]$$

(5) $$I_{L}\left(t\right)=p_{A,L}e^{-k_{1}t}+\frac{p_{B,L}k_{1}}{k_{2}-k_{1}}\left(e^{-k_{1}t}-e^{-k_{2}t}\right)+p_{C,L}\left[1+\frac{k_{1}e^{-k_{2}t}-k_{2}e^{-k_{1}t}}{k_{2}-k_{1}}\right]$$

where *p*_A,H_, *p*_B,H_ and *p*_C,H_ are the multiplication products of the initial concentration of A ([A]_o_) and the corresponding molar emission coefficients at λ_H_ (ε) (i.e., *p*_A,H_ = ε_A,H_[A]_o_, *p*_B,H_ = ε_B,H_[A]_o_ and *p*_C,H_ = ε_C,H_[A]_o_, and ε_A,H_, ε_B,H_ and ε_C,H_ being the molar emission coefficients at λ_H_ for A, B and C respectively). Similarly, *p*_A,L_, *p*_B,L_ and *p*_C,L_ are the products of the initial concentration of A ([A]_o_) and the corresponding molar emission coefficients at λ_L_ (ε) (i.e., *p*_A,L_ = ε_A,L_[A]_o_, *p*_B,L_ = ε_B,L_[A]_o_ and *p*_C,L_ = ε_C,L_[A]_o_, and ε_A,L_, ε_B,L_ and ε_C,L_ are the molar emission coefficients at λ_L_ for A, B and C respectively). 

By treating *p*_A,H_, *p*_B,H_, *p*_C,H_, *p*_A,L_, *p*_B,L_, *p*_C,L_, *k*_1_ and *k*_2_ as parameters and fitting Equations (4) and (5) to the experimental fluorescence intensity data, we found that *p*_B,H_,_,_ *p*_A,L_, and *p*_B,L_ do not contribute significantly to the corresponding observed intensities. Thus, Equations (4) and (5) could be reduced to Equations (6) and (7), and these equations were used to determine the values of k_1_ and k_2_.

(6) $$I_{H}\left(t\right)\approx p_{A,H}e^{-k_{1}t}+p_{C,H}\left[1+\frac{k_{1}e^{-k_{2}t}-k_{2}e^{-k_{1}t}}{k_{2}-k_{1}}\right]$$

(7) $$I_{L}\left(t\right)\approx p_{C,L}\left[1+\frac{k_{1}e^{-k_{2}t}-k_{2}e^{-k_{1}t}}{k_{2}-k_{1}}\right]$$

For the description of the variation of the corresponding fluorescence intensity of 1,5-DIN with time, an irreversible, consecutive “ABCD” (*A*→*B*→*C*→*D*) reaction model was used Equations (8) and (9).

(8) $$\begin{array}{l} I_{H}\left(t\right)\approx p_{B,H}\frac{k_{1}}{k_{2}-k_{1}}\left(e^{-k_{1}t}-e^{-k_{2}t}\right)+p_{D,H}[1-e^{-k_{1}t}-\frac{k_{1}}{k_{2}-k_{1}}\left(e^{-k_{1}t}-e^{-k_{2}t}\right)-\\ -\frac{k_{1}k_{2}}{\left(k_{1}-k_{2}\right)\left(k_{3}-k_{1}\right)}\left(e^{-k_{3}t}-e^{-k_{1}t}\right)-\frac{k_{1}k_{2}}{\left(k_{1}-k_{2}\right)(k_{2}-k_{3)}}\left(e^{-k_{3}t}-e^{-k_{2}t}\right)] \end{array}$$

(9) $$\begin{array}{l} I_{L}\left(t\right)\approx p_{D,L}[1-e^{-k_{1}t}-\frac{k_{1}}{k_{2}-k_{1}}\left(e^{-k_{1}t}-e^{-k_{2}t}\right)-\\ -\frac{k_{1}k_{2}}{\left(k_{1}-k_{2}\right)\left(k_{3}-k_{1}\right)}\left(e^{-k_{3}t}-e^{-k_{1}t}\right)-\frac{k_{1}k_{2}}{\left(k_{1}-k_{2}\right)\left(k_{2}-k_{3}\right)}\left(e^{-k_{3}t}-e^{-k_{2}t}\right)] \end{array}$$

where *k*_1_, *k*_2_ and *k*_3_ are the rate coefficients for the reaction *A*→*B*, *B*→*C* and *C*→*D*, respectively.

The values of *k*_1_, *k*_2_ and *k*_3_ were obtained by fitting Equations (8) and (9) to the experimental fluorescence intensity curves. For fitting of the parameters of Equations (6)–(9) to the experimental kinetic data and thus to determine the corresponding rate coefficients, a home-made parameter estimation software based on the Gauss–Newton–Marquardt procedure was applied [18]. 

## 3. Results and Discussion

In order to study the effect of the co-solvent and the solvent composition on the kinetics of the reaction of 1,5-isocyanoaminonaphthalene (1,5-ICAN) with water and Hg(II) ions, different aprotic (tetrahydrofuran, acetonitrile, N,N-dimethylformamide) and protic co-solvents (ethanol, 2-propanol) were used. Furthermore, to obtain pseudo first-order kinetics, water and HgCl_2_ were used in high excess to the fluorophore 1,5-ICAN (by several orders of magnitude) and the reactions were followed by recording the emission spectra as a function of time. The reactions were started by adding 1,5-ICAN to the solution of HgCl_2_.

### 3.1. Reactions in Water/Aprotic Solvent Mixtures

**Figure 1:**
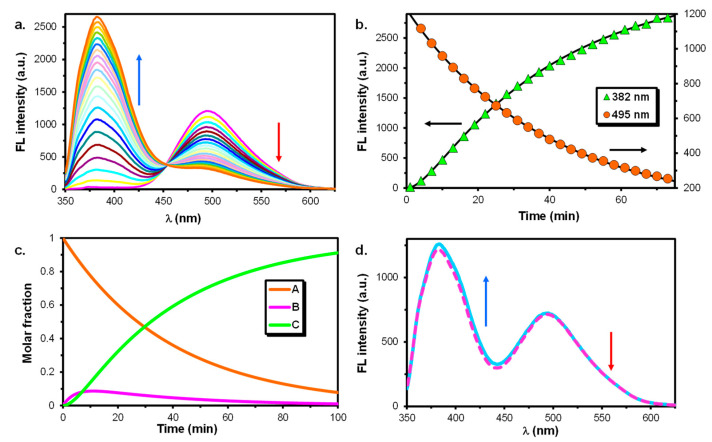
The evolution of the emission spectra during the reaction of 1,5-ICAN with water and HgCl_2_ in the mixture of THF/water (**a**). The variations of the fluorescence intensities at λ = 382 nm and 495 nm recorded at 60 s intervals. The symbols show the experimental data, while the solid lines represent the fitted curves by Equations (4) and (5) (**b**). The change of the molar fractions for the components A, B and C calculated by Equations (4) and (5) using the parameters obtained from fitting (**c**). Measured (solid line) and calculated (dashed line) emission spectra at 25 min reaction time (**d**). Experimental conditions: [H_2_O] = 27.8 mol/L (~ 50% (V/V)), [1,5-ICAN] = 2.5 × 10^−6^ mol/L, [HgCl_2_] = 1.2 × 10^−3^ mol/L and T = 25 °C.

The change of the emission spectra in a water/tetrahydrofuran (THF) mixture is shown in Figure 1a. 

**Scheme 1:**
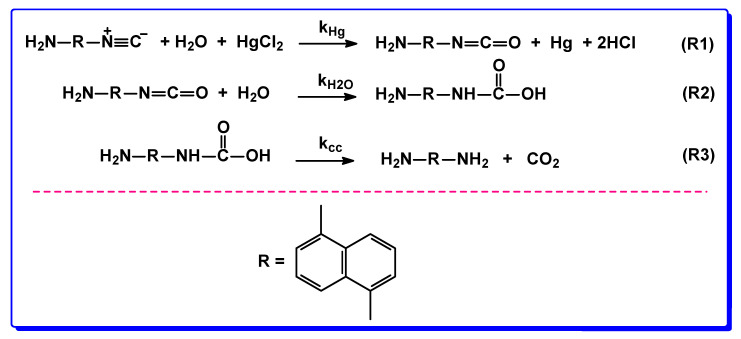
Reactions of 1,5-ICAN with water (R1-R3) and HgCl_2_.

As seen in Figure 1a, the emission intensity maximum at λ = 495 nm decreases, while at λ = 382, it increases continuously and the spectra show an isoemissive point at λ = 450 nm suggesting a simple A→B reaction. Moreover, as it is also evident from Figure 1b, the formation of the product of the reaction reveals a short “induction period”, a simple exponential fitting to the experimental curves gave no acceptable fitting, and the rate of consumption of 1,5-ICAN and that of the formation of the product are considerably different. The variation of the corresponding fluorescence intensities with time can adequately be described by a consecutive, first-order, “*A*→*B*→*C*” reaction model (see Equations (1)–(5) in the Experimental Section) as demonstrated in Figure 1b. In this reaction model, “*A*” represents the starting 1,5-ICAN and “*C*” the product 1,5-diaminonaphthalene (1,5-DAN). Based on the distribution of the starting *A*, the intermediate *B* and the product *C* (Figure 1c) determined from fitting, the corresponding emission spectra were also calculated. As seen in Figure 1d, there is a good agreement between the experimental and calculated spectra. Furthermore, the fact that the reaction product is 1,5-DAN has been unambiguously proved by comparing the fluorescence spectrum of the product and that of 1,5-DAN. In addition, the formation of 1,5-DAN was also supported by ESI-MS measurements (see later) and this finding is in line with what we observed earlier in water in the absence of co-solvents [14]. It was found that the irreversible, consecutive “*A*→*B*→*C*” reaction model is valid for all the systems containing aprotic co-solvents (good fitting was found in each case) and the product of the reaction is 1,5-DAN independently of the composition of the water/aprotic co-solvent systems. The proposed main reaction paths for the reaction of 1,5-ICAN with water and HgCl_2_ are summarized in Scheme 1.

**Figure 2:**
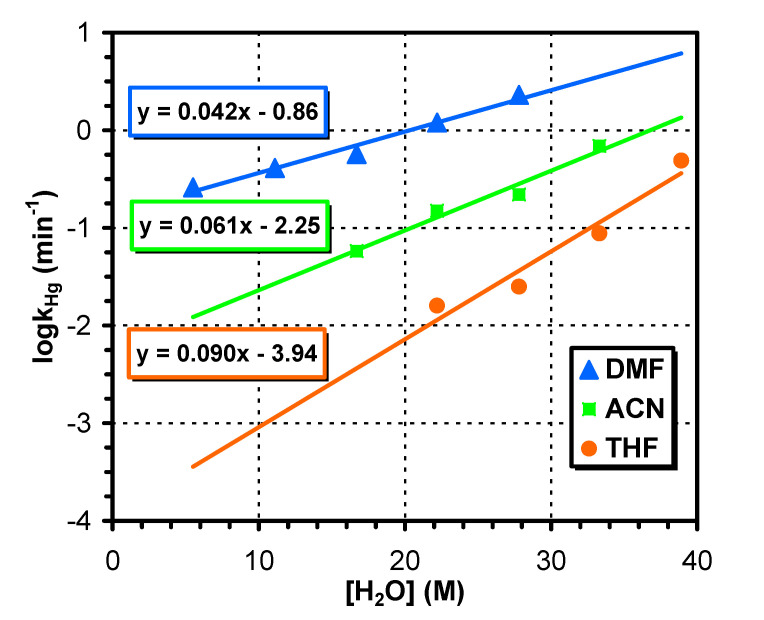
Variation of the logk_Hg_ values with the water concentration for the aprotic co-solvents. Experimental conditions: [1,5-ICAN] = 2.5 × 10^−6^ mol/L, [HgCl_2_] = 1.2 × 10^−3^ mol/L and T = 25 °C.

According to Scheme 1, isocyanate as an intermediate is formed in R1. R1 is likely to be a net reaction consisting of various elementary steps. Formations of the intermediate isocyanate from isonitrile in the presence of water and Hg(II)-ions have been also reported [19,20,21]. The isocyanate can react further with water to form carbamic acid in R2 followed by its decomposition to yield the amine derivative in R3. The reactions R2 and R3 are well-known reactions in polyurethane chemistry that are used in the process of making polyurethane foams [22,23,24]. Furthermore, it is also assumed that the rate-determining step to form amine from isocyanate is due to the reaction of isocyanate with water (R2) and the decomposition of the formed carbamic acid to amine is a relatively fast process. Hence, in this scenario, the intermediate “*B*” in the consecutive “*A*→*B*→*C*” reaction model represents the isocyanate formed in R1. The corresponding pseudo first-order rate coefficients k_Hg_ (k_1_) and k_H2O_ (k_2_) were obtained by fitting Equations (4) and (5) to the experimental fluorescence curves as detailed in the Experimental Section. To visualize the trends in the changes of the k_Hg_ values, the variations of the logarithm of k_Hg_ values with the solvent compositions are plotted in Figure 2, while the values of k_Hg_ and k_H2O_ are compiled in Table 1.

From Figure 2 and the data of Table 1, k_Hg_ values increase unambiguously with the water content in each case, and they also increase in the order of THF < ACN < DMF at the same water concentration (see Table 1, indicated by dashed line). It is also evident that the extent of the increase in the k_Hg_ values as going from THF to DMF at 27.8 M water content is similar to the case when [H_2_O] = 22.2 M. Furthermore, it can also be surmised from the data presented in Figure 2 and Table 1 that k_Hg_ values increase more steeply (i.e., with higher slope) with the water concentration for the less polar THF (dielectric constant = 7.6, [25]) than for the other two more polar solvents (dielectric constant = 36.7 and 37.5 for DMF and ACN, respectively, [25]) and the slope of log k_Hg_ versus [H_2_O] decreases in the order of THF > ACN > DMF as judged from the corresponding slopes in Figure 2 (i.e., 0.090, 0.061 and 0.04, respectively). The fact that k_Hg_ values increase with water concentration may not be so surprising since water molecules are likely to be involved in the rate-determining reaction step leading to the formation of isocyanate intermediate (R1). However, water can act not only as a reactant (specific interaction) but it also increases the overall polarity of the reaction medium (non-specific interaction), which likely favors reaction R1 to take place. Hence, the increase in k_Hg_ values with the increasing water concentration may be the result of the specific and non-specific interactions. Furthermore, it can also be rationalized that the steepest increase in the k_Hg_ values is in the case of THF since the highest relative change in the polarity of a solvent mixture with the increasing water content can be expected in the case of a co-solvent of low polarity. 

Stemming also from the data of Table 1, only moderate changes are seen for k_H2O_. Thus, the k_H2O_ values vary with the increasing water concentration less significantly than the k_Hg_ values. For example, for can, a *ca* 12-fold increase in the value of k_Hg_ can be observed as going from [H_2_O] = 16.7 M to 33.3 M, while the corresponding k_H2O_ values shows only a 4-fold increase. 

### 3.2. Reactions in Water/Protic Solvent Mixtures

**Figure 3:**
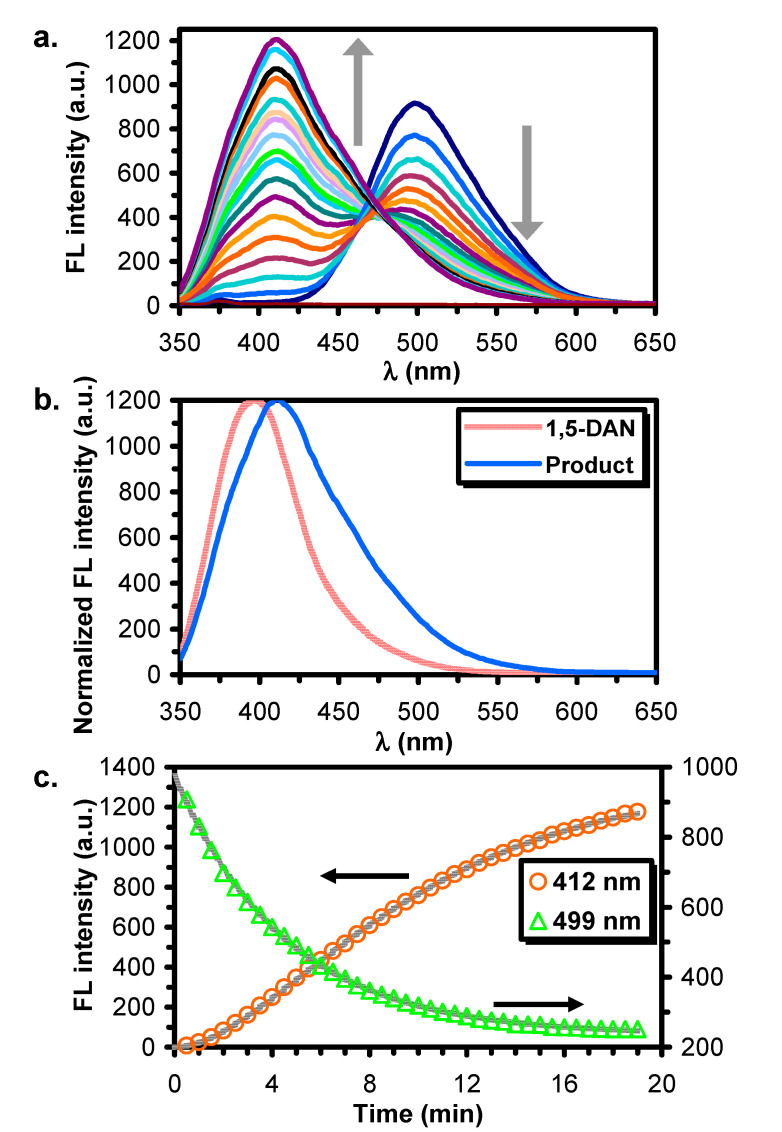
Evolution of the emission spectra during the reaction of 1,5-ICAN with the mixture of water/*i*PrOH and HgCl_2_ (**a**). The normalized fluorescence (FL) intensity of 1,5-DAN and that of the final reaction product (**b**) and the variations of the fluorescence intensities recorded at λ = 412 nm and 499 nm at 30 s intervals (**c**). The symbols show the experimental data, while the black solid lines represent the fitted curves by Equations (4) and (5). Experimental conditions: [H_2_O] = 27.8 mol/L (~50% (V/V)), [1,5-ICAN] = 2.5 × 10^−6^ mol/L, [HgCl_2_] = 1.2 × 10^−3^ mol/L and T = 25 °C.

The 1,5-ICAN in the presence of HgCl_2_ in water/protic solvents behaves similarly to those in water/aprotic co-solvents, i.e., the emission at around 500 nm decreases continuously in parallel with the fluorescence intensity increase at around 410–420 nm as demonstrated in Figure 3a for the water/2-propanol (*i*PrOH) mixture. 

**Figure 4:**
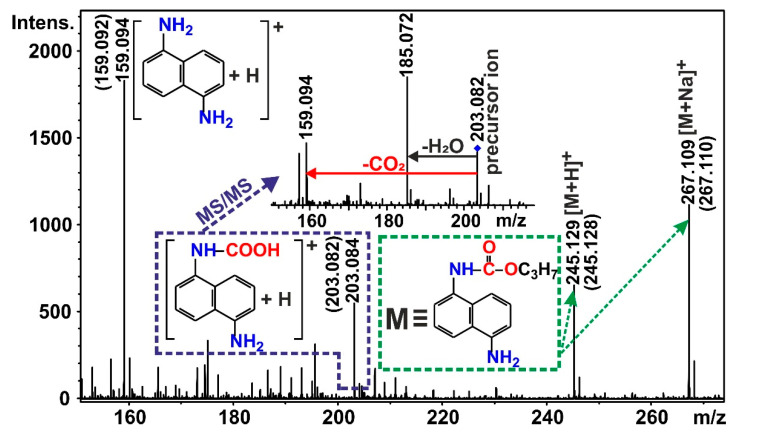
ESI-MS spectrum of the reaction mixture obtained in the reaction of 1,5-ICAN with the mixture of water/*i*PrOH and HgCl_2_. The numbers in brackets are the calculated *m/z* values. The inset shows the MS/MS spectrum of the protonated (5-aminonaphthalen-1-yl)carbamic acid at *m/z* 203.

However, comparing the emission spectrum of the product to that of the 1,5-DAN (Figure 3b), it is now evident that the final product is not 1,5-DAN (or not exclusively). Interestingly, the “ABC” reaction model is still capable of describing the fluorescence intensity variations with time as evidenced by Figure 3c. In order to determine the reaction product(s) formed in the water/*i*PrOH mixture, ESI-MS measurements were performed. The reaction mixtures for ESI-MS were prepared similarly to those for the fluorescence experiments, but in the ESI-MS experiments, 1,5-ICAN was applied at a higher concentration by one order of magnitude. The ESI-MS spectrum of the reaction mixture obtained in a water/*i*PrOH solution is shown in Figure 4.

**Scheme 2:**
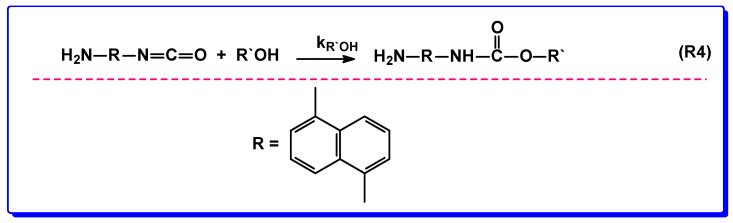
Reaction of 1,5-ICAN with alcohols.

As indicated in Figure 4, the ESI-MS spectrum reveals the presence of the protonated 1,5-DAN, (5-aminonaphthalen-1-yl)carbamic acid and isopropyl (5-aminonaphthalen-1-yl)carbamate (urethane) at *m/z* 159, 203 and 245, respectively. According to Figure 4, the measured and the calculated *m/z* (based on the corresponding composition) values are also in good agreement. To further confirm the presence of (5-aminonaphthalen-1-yl)carbamic acid, ions at *m/z* 203 were subjected to MS/MS. As seen in the Figure 4 inset, the MS/MS spectrum displays subsequent losses of H_2_O and CO_2_ molecules, supporting the presence of carbamic acid in the reaction mixture. Indeed, the formation of (5-aminonaphthalen-1-yl)carbamic acid and isopropyl (5-aminonaphthalen-1-yl)carbamate confirm the formation of isocyanate groups. The isocyanates can react with water to yield 1,5-DAN through the decomposition of the carbamic acids (R2 and R3 in Scheme 1) in the presence of water and protic co-solvents like 2-propanol or ethanol. On the other hand, they can also react with alcohols to form carbamate derivatives as depicted in Scheme 2. Thus, the findings that carbamate and carbamic acid are present in the reaction mixture support the formation of isocyanate as an intermediate during the reaction.

**Figure 5:**
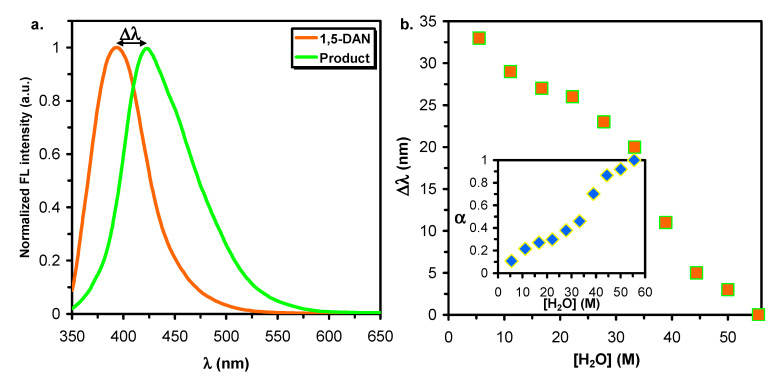
The normalized fluorescence (FL) spectra (**a**) and the difference (Δλ)* in the emission maxima of 1,5-DAN and that of the product(s) formed in the reaction of 1,5-ICAN in the mixture of EtOH/water and HgCl_2_ (**b**). The inset shows the variation of the α values with the water concentration in the water/EtOH mixture. Experimental conditions: [1,5-ICAN] = 2.5 × 10^−6^ mol/L, [HgCl_2_] = 1.2 × 10^−3^ mol/L and T = 25 °C. * The Δλ values indicated were obtained using the same solvent composition.

This latter reaction, i.e., the nucleophilic addition of alcohols to isocyanates, also forms the basis of industrial polyurethane (PUR) manufacturing [18]. Thus, it can be concluded that the conversion of the isocyano group to amine and carbamate moieties takes place simultaneously in water/alcohol mixtures. In addition, looking at the broad fluorescence band belonging to both 1,5-DAN and ethyl-(5-aminonaphthalen-1-yl)carbamate, it is evident that they do not appear as separate bands, rather they are superimposed. Consequently, it is likely a composite band and only a shift of the emission maximum with the increasing water content towards that of the 1,5-DAN can be observed as demonstrated in Figure 5. 

As seen in Figure 5a, there is a considerable difference (Δλ) in the λ_max_ values of 1,5-DAN and the product of the reaction. On the other hand, it is also evident from Figure 5b that the Δλ value decreases with the increasing water concentration. This latter finding is due, most probably, to the fact that at higher water content, 1,5-DAN is formed in a higher amount with respect to the carbamate derivative. If we assume that R2 and R4 reactions take place in parallel, and the formed carbamic acid decomposes relatively fast (R3), the molar ratio of 1,5-DAN/carbamate is equal to the ratio of the corresponding pseudo rate coefficients, i.e., k_H2O_/k_R`OH_ (see reactions R2 and R4 in Scheme 1 and Scheme 2, respectively). Considering that both fluorescence bands are relatively broad and can be described by a Gaussian-like curve, the maximum λ value for the peak consisting of fluorescence bands of 1,5-DAN (DAN) and carbamate derivative (CAR) can be approximated by Equation (10) as:

(10) $$\lambda =Y_{\mathrm{DAN}}\lambda_{\mathrm{DAN},\max}+Y_{\mathrm{CAR}}\lambda_{\mathrm{CAR},\max}$$

where *Y*_DAN_ and *Y*_CAR_ are the fluorescence intensity fraction of 1,5-DAN and the carbamate derivative contributing to the intensity of the composite peak, respectively, *Y*_DAN_ = ε_DAN_C_DAN_/(ε_DAN_C_DAN_+ε_CAR_C_CAR_) and *Y*_CAR_ = 1 − *Y*_DAN_, where ε_DAN_ and ε_CAR_ are the molar emission coefficients, and C_DAN_ and C_CAR_ are the concentrations of 1,5-DAN and the carbamate derivative, respectively. 

Furthermore, if we assume that ε_DAN_ ≈ ε_CAR_ is valid then *Y*_DAN_ and *Y*_CAR_ values refer to the corresponding molar fractions, i.e., *X*_DAN_ and *X*_CAR_, and Equation (10) reduces to Equation (11).

(11) $$\Delta \lambda =\left(1-X_{\mathrm{DAN}}\right)\left(\lambda_{\mathrm{CAR},\max}-\lambda_{\mathrm{DAN},\max}\right)$$

where Δ*λ* is the wavelength difference between the emission maximum of the product and that of the 1,5-DAN in the same solvent composition. The value of *λ*_CAR,max_ in EtOH was estimated by the extrapolation of *λ*_CAR,max_ values to zero water concentration. 

Then the fraction of k_H2O_ to *k*_H2O_ + *k*_R`OH_ (α) can be given by Equation (12) as:

(12) $$\alpha =\frac{k_{H2O}}{k_{H2O}+k_{R\text{`}\mathrm{OH}}}=1-\frac{\Delta \lambda }{\lambda_{\mathrm{CAR},\max}-\lambda_{\mathrm{DAN},\max}}$$

As seen in Figure 5b and its inset, as the water concentration is increased, the value of Δλ decreases, and α also increases with the water concentration. These findings indicate that in a mixture of higher water content, the formation of 1,5-DAN is preferred, while in solvents with high alcohol concentration, the reaction yielding a carbamate derivative dominates over 1,5-DAN formation. 

**Figure 6:**
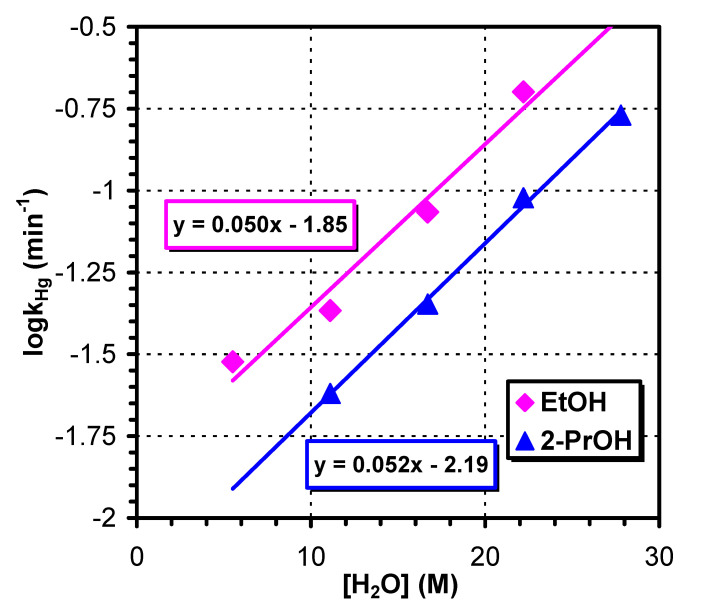
Variation of the logk_Hg_ values with the water concentration for the protic co-solvents. Experimental conditions: [1,5-ICAN] = 2.5 × 10^−6^ mol/L, [HgCl_2_] = 1.2 × 10^−3^ mol/L and T = 25 °C.

Furthermore, as it turns out from Figure 6 and the data of Table 2, k_Hg_ values increase considerably with the increasing water concentration. The relative value of k_Hg_ in EtOH to those obtained in solvent mixtures containing *i*PrOH does not change significantly, i.e., the k_Hg,EtOH_/k_Hg,*i*PrOH_ ratio is about 2 (see the data indicated by a dashed frame in Table 2). Thus, the data also reveal that reactions take place faster in solvent mixtures with EtOH than in those with *i*PrOH of the same composition. On the contrary, the values of k_H2O_ + k_R`OH_ do not vary significantly, but the fraction of the reactions leading to the formation of 1,5-DAN and the carbamate derivative change markedly as we have shown above (Figure 5b inset). The higher k_Hg_ and k_H2O_ + k_R`OH_ values in EtOH/water with respect to those obtained in *i*PrOH/water mixtures (dielectric constant = 18.3, [25]) can most likely be attributed to the higher polarity of the water/EtOH (dielectric constant = 22.4, [25]) solution than that of the water/*i*PrOH system.

### 3.3. Reaction of 1,5-DIN with Water and HgCl_2_

**Figure 7:**
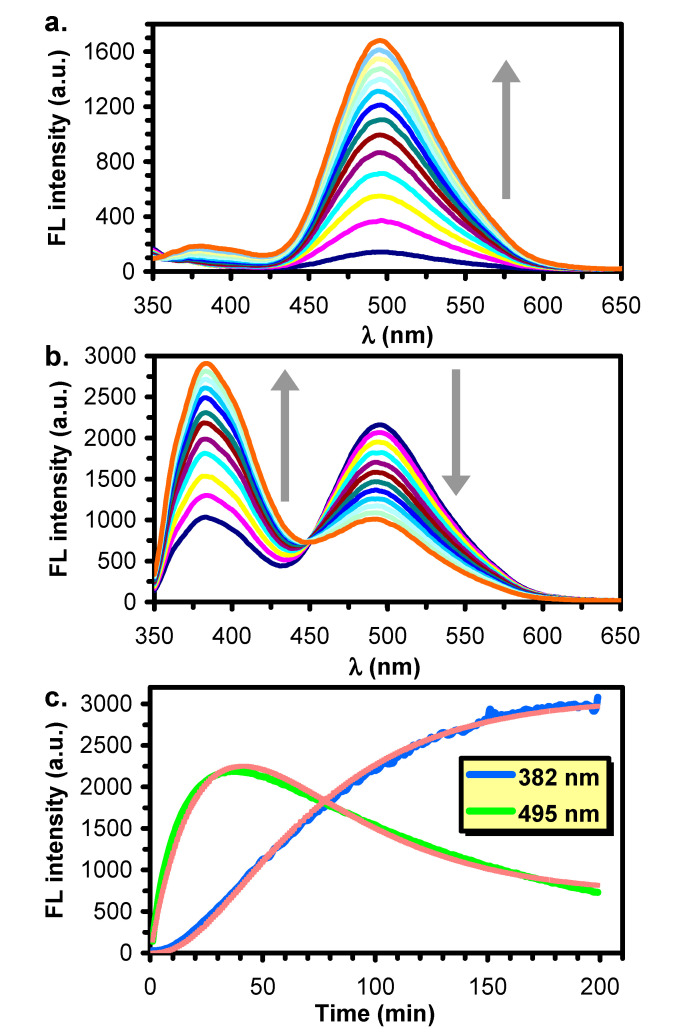
The evolutions of the emission spectra during the reaction of 1,5-DIN with the mixture of THF/water and HgCl_2_ in the time interval 0–27 min (**a**) and 90–210 min (**b**). The variations of the fluorescence intensities at λ = 382 nm and 495 nm recorded at 60 s intervals. The blue and the green solid lines represent the measured fluorescence intensities at λ = 382 nm and 495 nm, respectively, and the red solid lines are the fitted curves using Equations (8) and (9) (**c**). Experimental conditions: [H_2_O] = 27.8 mol/L (~50% (*V*/*V*)), [1,5-DIN] = 2.5 × 10^−6^ mol/L, [HgCl_2_] = 1.2 × 10^−3^ mol/L and T = 25 °C.

The kinetics of the conversion of two isocyano groups to diamine in the presence of water and HgCl_2_ was also studied. In this experiment, the reaction of 1,5-diisocyanonaphthalene (1,5-DIN) was also followed by fluorometry similarly to that of 1,5-ICAN. The changes of the fluorescence spectra of the reaction mixture containing 1,5-DIN are shown in Figure 7a,b.

**Scheme 3:**
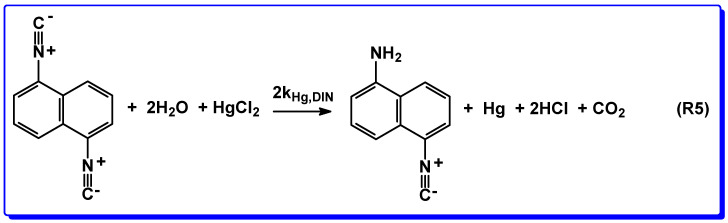
Reaction of 1,5-DIN with water and HgCl_2_. Factor 2 in the rate-coefficient (k_Hg,DIN_) expresses that there are two isocyano groups in the 1,5-DIN, which supposedly have equal reactivity.

As can be seen in Figure 7a,b, the emission band at 500 nm, characteristic of 1,5-ICAN appeared and the intensity of this band continuously increases until it reaches a maximum value followed by a slow decrease in the intensity. In addition, parallel with the intensity decrease at 500 nm, a new band at around 382 nm is formed, whose intensity increases steadily. This finding can be interpreted in terms of the following: the initial 1,5-DIN is not fluorescent and conversion of one of its isocyano groups to amine takes place to yield fluorescent 1,5-ICAN as shown in Scheme 3 (R5). 

This process is accompanied by an initial steep increase in the intensity of the band at 500 nm followed by a moderate decrease. In parallel with this process, due to the conversion of the remaining isocyano group to amine to afford the formation of 1,5-ICAN via reactions R1, R2 and R3 (Scheme 1), an increase in the intensity of the band at around 380 nm can be observed. By extending the “ABC” model with the reaction R5 and using the pseudo first-order rate coefficients determined independently for the 1,5-ICAN system in a water/THF mixture at [H_2_O] = 27.8 mol/L, i.e., k_Hg_ (k_1_) = 0.025 and k_H2O_ (k_2_) = 0.22 min^−1^ (see Table 1), and by fitting Equations (8) and (9), good matches were found as can be seen in Figure 7c (the errors of fittings were within ±2%). From the fitting, k_Hg,DIN_ was determined to be 0.015 min^−1^, which is lower than k_Hg_, indicating a positive substitution effect of the amino group with respect to the isocyano moiety.

## 4. Conclusions

The reactions of 1,5-ICAN with water in the presence of different protic and aprotic co-solvents and HgCl_2_ were studied by steady-state fluorescence measurements. It was found that using aprotic co-solvents, complete transformation of the isocyano moiety to amino group took place, i.e., formation of 1,5-DAN was observed. Interestingly, with protic co-solvents such as EtOH or *i*PrOH, in addition to 1,5-DAN, (5-aminonaphthalen-1-yl)carbamate was formed by the nucleophilic addition reaction of the intermediate isocyanate with alcohol. The formations of 1,5-DAN and the carbamate derivative were also confirmed by ESI-MS and ESI-MS/MS measurements. For the description of variations of the observed fluorescence properties, an irreversible, consecutive “ABC” reaction model was proposed. By fitting the parameters of the model to the experimental data we were able to determine the corresponding pseudo first-order rate coefficients. The rate coefficients were found to increase with water concentration and in the order of THF < ACN < DMF for aprotic co-solvents, while for protic co-solvents the order varied as EtOH > *i*PrOH. However, the rate coefficients for the reaction leading to the formation of the isocyanate intermediate increased more steeply in less polar co-solvents. Extending the proposed kinetic model with an additional reaction step, the variations of fluorescence intensities with time for the 1,5-DIN in water/THF mixture could also be adequately described. Furthermore, our kinetic experiments shed more light on the fact that the aprotic solvents, in the presence of water, do not alter significantly the efficiency of the Hg(II)-ion detection. However, the response time can vary with the polarity of the solution and a practical consequence of this finding is that to obtain a short response time, application of a more polar co-solvent or co-solvent system (if necessary) is preferred. Furthermore, protic co-solvents like alcohols do not quench, and just give rise to a 20–40 nm red-shift in the emission properties of the product formed in the presence of Hg(II) ions. However, it is still a large wavelength difference between 1,5-ICAN and 1,5-DAN and/or the carbamate derivative for making the ratiometric probing of Hg(II) in aqueous medium possible. In addition, it is reasonable to assume that these findings in connection with the 1,5-ICAN and its properties can also be utilized during the development of other analytical fluorophores containing isocyano group(s) as well.

## Figures and Tables

**Table 1:** The pseudo first-order rate coefficients for the reaction of 1,5-ICAN with water and HgCl_2_ in different water/aprotic co-solvents mixtures. The errors of the obtained rate coefficients were within ±10%. Experimental conditions: [1,5-ICAN] = 2.5 × 10^−6^ mol/L, [HgCl_2_] = 1.2 × 10^−3^ mol/L and T = 25 °C.

	k (min^−1^)	[H_2_O] (mol/L)						
		5.5	11.1	16.7	22.2	27.8	33.3	38.9
THF	k_Hg_ (k_1_)	*-*	*-*	*-*	*0.016*	*0.025*	*0.088*	*0.49*
	k_H2O_ (k_2_)	*-*	*-*	*-*	*0.10*	*0.22*	*0.20*	*0.62*
ACN	k_Hg_ (k_1_)	*-*	*-*	*0.058*	*0.15*	*0.22*	*0.69*	*-*
	k_H2O_ (k_2_)	*-*	*-*	*0.070*	*0.099*	*0.16*	*0.28*	*-*
DMF	k_Hg_ (k_1_)	*0.26*	*0.41*	*0.57*	*1.2*	*2.3*	*-*	*-*
	k_H2O_ (k_2_)	*1.3*	*1.7*	*1.8*	*2.7*	*2.5*	*-*	*-*

**Table 2:** The pseudo first-order rate coefficients for the reaction of 1,5-ICAN with water in different water/protic co-solvents mixtures and HgCl_2_. The errors of the obtained rate coefficients were within ±10%. Experimental conditions: [1,5-ICAN] = 2.5 × 10^−6^ mol/L, [HgCl_2_] = 1.2 × 10^−3^ mol/L and T = 25 °C.

	k (min^−1^)	[H_2_O] (mol/L)				
		5.5	11.1	16.7	22.2	27.8
EtOH	k_Hg_ (k_1_)	*0.030*	*0.043*	*0.086*	*0.20*	*-*
	k_H2O_ + k_R`OH_ (k_2_)	*0.43*	*0.40*	*0.63*	*0.68*	*-*
*i*PrOH	k_Hg_ (k_1_)	*-*	*0.024*	*0.045*	*0.095*	*0.17*
	k_H2O_ + k_R`OH_ (k_2_)	*-*	*0.091*	*0.14*	*0.12*	*0.18*
